# Substrate Composition Modulates Agri-Food Waste Bioconversion by Yellow Mealworm (*Tenebrio molitor*) Larvae Under Dynamic Feeding

**DOI:** 10.3390/insects17070692

**Published:** 2026-07-03

**Authors:** Jingtao Liu, Chenyang Li, Peng Wang, Hongyue Wang, Chuxuan Nie, Rongrong Zhao, Jiaoxin Xie

**Affiliations:** 1 Dazhou Key Laboratory of Agricultural Resources Development and Ecological Conservation in Daba Mountain, Sichuan University of Arts and Science, Dazhou 635000, China; knighteva@163.com (J.L.); wanghongyue789@163.com (H.W.); niechuxuan7149@163.com (C.N.); zhaorongrong625@163.com (R.Z.); 2 College of Animal Science, Shanxi Agricultural University, Taigu 030801, China; pploveanimal@gmail.com (C.L.); w18534271561@163.com (P.W.)

**Keywords:** *Tenebrio molitor*, substrate composition, agri-food waste, dynamic feeding, bioconversion, okara, kitchen waste, nutritional remodelling, heavy metals

## Abstract

Yellow mealworm (*Tenebrio molitor*) larvae can convert low-value organic residues into insect biomass, but their performance depends on substrate composition and feeding strategy. We evaluated vegetable wastes, okara–wheat–bran diets and kitchen waste–wheat–bran mixtures under a dynamic feeding regime. Ingredient and proximate compositions were determined, and larval growth, fresh-weight-based waste reduction (WR), bioconversion rate (BCR) and feed conversion efficiency (FCE), pupal output, nutritional composition and heavy metal contents were assessed. Among vegetable wastes, potato showed the highest numerical WR (95.18 ± 0.73%) and relatively high BCR and FCE, whereas pumpkin produced the most pupae (109.00 ± 8.62 per replicate). Cabbage showed reduced biomass conversion and pupal output. In the okara trial, 40% okara showed the highest numerical WR (75.88 ± 0.39%), 10% okara maximized FCE (15.83 ± 0.38%) and 20% okara produced the greatest pupal output. Principal component analysis (PCA) indicated treatment-specific conversion and developmental patterns, but was interpreted as an exploratory association analysis rather than a causal model. Kitchen waste mixtures increased the relative fat proportion and reduced the relative protein proportion of larvae; the 3:2 mixture increased crude fat to 37.90 ± 0.22% while retaining 57.39 ± 0.40% crude protein. Higher kitchen waste inclusion was associated with greater larval arsenic (As) content, reaching 0.803 ± 0.001 mg/kg in the 5:2 treatment. Substrate composition should therefore be optimized together with conversion efficiency, product quality and safety screening.

## Simple Summary

Yellow mealworm (*Tenebrio molitor*; *T. molitor*) larvae can convert organic residues into insect biomass, but their performance depends strongly on substrate composition and feeding strategy. This study evaluates vegetable wastes, okara–wheat–bran diets and kitchen waste–wheat–bran mixtures under a dynamic feeding regime. Ingredient composition and proximate composition are included to clarify the nutritional basis of each treatment. Among the vegetable wastes tested, potato, zucchini and pumpkin showed greater potential, whereas cabbage showed reduced biomass conversion and lower pupal output despite moderate waste reduction. Okara substitution produced target-dependent responses: higher okara levels favoured waste reduction, lower levels improved feed conversion efficiency and 20% okara supported greater pupal output. Kitchen waste mixtures increased the relative fat proportion of larvae but reduced the relative crude protein proportion, and higher kitchen waste inclusion was associated with greater larval arsenic content in the preliminary safety screen. These findings indicate that *T. molitor*-based agri-food waste valorization should balance substrate composition, waste reduction, larval production, nutritional quality and safety screening.

## 1. Introduction

Okara, kitchen waste and vegetable waste are common high-moisture agri-food by-products. Okara contains protein, dietary fibre, lipids and carbohydrates but spoils rapidly, whereas kitchen waste is heterogeneous and vegetable waste often has high moisture and low dry matter [1,2,3]. Conventional disposal routes do not always combine organic matter reduction with recovery of proteins, lipids and other products; insect bioconversion therefore offers a route for upgrading these residues into biomass, feed ingredients and frass [4,5].

Insect bioconversion can transform low-value substrates into protein- and lipid-rich biomass [4,5]. Yellow mealworm (*Tenebrio molitor*; *T. molitor*) is attractive for feed development because of its established rearing systems, relatively high protein content and capacity to use plant-based by-products [6,7]. However, *T. molitor* performance is strongly substrate-dependent: agro-industrial diets affect growth, digestibility, body composition, gut microbiota, fatty acid profile and developmental time [8,9,10,11].

Single-substrate feeding may be limited when moisture, fibre or nutrient balance does not meet larval requirements [12,13,14]. *T. molitor* larvae show age-dependent macronutrient selection, and dietary protein, carbohydrate and energy ratios affect yield and feed conversion [15,16,17]. Blending substrates can mitigate nutritional or physical limitations; for example, okara–kitchen waste mixtures improve black soldier fly performance, whereas different *T. molitor* by-products alter growth and composition [1,12,13,14].

Beyond conversion efficiency, nutritional composition and safety determine whether insect biomass is suitable as a feed resource. Agricultural sidestreams and by-product substrates can change *T. molitor* nutrient profile, mineral composition, heavy metal uptake and product safety [18,19,20]. For complex substrates such as kitchen waste, heavy metals and microbial hazards should therefore be considered together with conversion and nutritional value [7,21].

Although okara and kitchen waste mixtures have been studied more extensively in black soldier fly systems, the response of *T. molitor* to vegetable waste, okara and kitchen waste within a single evaluation framework remains insufficiently characterized [1,7,8,22]. Previous *T. molitor* studies have usually focused on one by-product class, one formulated diet or one performance endpoint, whereas direct comparison of vegetable wastes, okara substitution and kitchen waste mixtures under a dynamic feeding strategy is still limited [8,9,10,18,19,20]. In this study, *T. molitor* larvae were reared under a dynamic feeding regime to compare the effects of different vegetable wastes and okara substitution levels on fresh-weight-based waste reduction (WR), bioconversion rate (BCR), feed conversion efficiency (FCE) and pupal output. We also evaluated the effects of kitchen waste proportion on larval crude protein, crude fat and arsenic (As), cadmium (Cd), mercury (Hg) and lead (Pb) contents. By integrating growth dynamics, conversion efficiency, developmental output, nutritional composition, substrate composition and heavy metal screening, this study provides a broader experimental framework for assessing *T. molitor* in multisource agri-food waste valorization.

## 2. Materials and Methods

### 2.1. Insects

Yellow mealworm (*T. molitor*) larvae were obtained from a laboratory colony maintained at the Apiculture Laboratory of Shanxi Agricultural University. The colony was originally established from a commercial source and had been reared continuously in the laboratory for more than three years. Insects were kept in climate chambers at 28 ± 1 °C, 60 ± 5% relative humidity and a 16:8 h light:dark photoperiod. The stock colony was maintained on wheat bran and was regularly supplemented with fresh carrot as a moisture source and minor nutrient supplement. Before the experiments, actively feeding late-instar larvae with normal body colour and similar size were selected. Individuals showing poor movement, body damage, abnormal moulting or obviously small size were excluded to reduce variation in initial developmental status.

### 2.2. Substrate Sources and Preparation

Vegetable waste, okara and kitchen waste were selected as representative agri-food organic waste substrates. The vegetable treatments included pumpkin, carrot, potato, zucchini and cabbage, with wheat bran as the control substrate. Visible mouldy, rotten and non-edible materials were removed before feeding. Vegetable materials were washed, chopped into pieces suitable for larval feeding and thoroughly mixed. Okara, a food-processing by-product, was mixed with wheat bran to test how substitution with a wet by-product affected larval conversion and pupal output. Kitchen waste was used to evaluate how a complex urban organic waste stream affected larval nutritional composition and heavy metal accumulation. Non-food materials such as plastic, bones and packaging were removed from the kitchen waste before use; the remaining material was ground, homogenized and mixed with wheat bran at the designed ratios.

### 2.3. Experimental Design and Dynamic Feeding Strategy

All feeding trials used a dynamic feeding strategy. Each treatment had three biological replicates. The vegetable waste and okara substitution trials lasted 25 d. In the vegetable waste trial, each replicate was initiated with 59.46–66.44 g of late-instar larvae, corresponding to an estimated 316–678 larvae per replicate based on the 30-larva subsample weight. In the okara substitution trial, each replicate was initiated with 30.01–30.09 g of late-instar larvae, corresponding to approximately 288–352 larvae per replicate. The kitchen waste trial lasted 25 days before larval samples were collected for nutritional and heavy metal analyses. Larvae were fed daily, but the daily feed amount was recalculated every 5 days. During the first 5-day cycle, the daily feed amount was 20% of the initial total larval biomass in each replicate. At the end of each 5-day cycle, total larval biomass in each replicate was reweighed, and 20% of that biomass was used as the daily feed amount for the next 5-day cycle. This process was repeated until the end of the rearing period. The strategy allowed feed supply to follow changes in larval biomass and reduced the risk of feed shortage or excessive residue accumulation.

### 2.4. Vegetable Waste Feeding Trial

The vegetable waste trial included six treatments: pumpkin, carrot, potato, zucchini, cabbage and a wheat bran control. Each vegetable treatment contained 100% of the corresponding fresh vegetable material, whereas the control contained 100% wheat bran on a fresh-weight basis. Wheat bran contained 13.0% moisture and 87.0% dry matter; the vegetable substrates were high-moisture materials, with 79.3–94.8% moisture and 5.2–20.8% dry matter. Their crude protein, crude fat, ash and carbohydrate contents on a dry-matter basis were 7.9–23.2%, 0.4–6.1%, 5.4–11.1% and 59.7–84.3%, respectively. All treatments followed the dynamic feeding regime described above. During the trial, total larval biomass, residual feed, frass and dead larvae were recorded for each replicate. Pupae were collected throughout the rearing period, and pupal number and pupal weight were recorded. At the end of the experiment, cumulative feed input, cumulative residual feed, final larval biomass, total pupal number and total pupal weight were calculated to evaluate the effects of single vegetable substrates on *T. molitor* growth, conversion and developmental stability.

### 2.5. Okara Substitution Feeding Trial

For the okara substitution trial, wheat bran was used as the basal substrate. Four substitution levels were tested: 50%, 40%, 20% and 10% okara, corresponding to wheat bran:okara ratios of 50:50, 60:40, 80:20 and 90:10 on a fresh-weight basis. A 100% wheat bran treatment served as the control. Okara contained 78.0% moisture, 22.0% dry matter, 28.5% crude protein and 9.0% crude fat; the mixed okara diets contained 19.5–45.5% moisture, 54.5–80.5% dry matter, 17.6–19.6% crude protein, 4.0–4.9% crude fat, 5.3–5.6% ash and 70.2–72.8% carbohydrate. All treatments followed the same dynamic feeding strategy. The measurements and calculations were the same as those used in the vegetable waste trial, with individual larval weight also recorded to evaluate the effects of okara proportion on substrate use, biomass accumulation and pupal output.

### 2.6. Kitchen Waste Feeding Trial and Sample Collection

The kitchen waste trial was designed to evaluate the effects of a complex organic waste stream on larval nutritional composition and heavy metal accumulation. Three treatments were used: wheat bran control, kitchen waste:wheat bran = 3:2 and kitchen waste:wheat bran = 5:2. The 3:2 and 5:2 treatments corresponded to 60.0% and 71.4% kitchen waste on a fresh-weight basis, respectively. Kitchen waste contained 68.0% moisture, 32.0% dry matter, 17.5% crude protein and 21.7% crude fat; the 3:2 and 5:2 mixed diets contained 46.0% and 52.3% moisture, 54.0% and 47.7% dry matter, 17.4% crude protein, 10.2% and 12.4% crude fat, 5.0% and 4.8% ash, and 67.4% and 65.3% carbohydrate, respectively. All groups were reared under the same environmental conditions. At the end of the experiment, larval samples were collected from each treatment. Surface debris was removed before moisture, crude protein and crude fat analyses. Samples for heavy metal analysis were dried, ground and digested before determination of As, Cd, Hg and Pb contents.

### 2.7. Calculation of Conversion Efficiency Indices

Fresh-weight-based operational conversion indices were calculated according to the dynamic feeding strategy, including WR, BCR and FCE. For each 5-day cycle, total feed input equalled larval biomass at the beginning of that cycle because the daily feed input was 20% of that biomass for five consecutive days. Cumulative feed input was calculated as the sum of all cycle inputs. The indices were calculated as follows:

WR (%) = (*F* − *R*)/*F* × 100 (1)

BCR (%) = (*B_f_* − *B_i_*)/*F* × 100 (2)

FCE (%) = (*B_f_* − *B_i_*)/(*F* − *R*) × 100 (3)

where *F* is the cumulative feed input, *R* is the cumulative residual feed, *B_i_* is the initial larval fresh weight and *B_f_* is the final harvested biomass. In the vegetable trial, *B_f_* was the final larval fresh weight. In the okara trial, *B_f_* included final larval fresh weight plus harvested pupal fresh weight. Because the conversion indices were calculated on a fresh-weight basis, they are reported as operational conversion indices and interpreted together with substrate proximate composition.

### 2.8. Nutritional Composition Analysis

Larval moisture content was determined by oven drying. A known amount of larval sample was dried at 100 °C for 2 h, and the moisture content was calculated from the mass loss. Crude fat content was measured by Soxhlet extraction using petroleum ether (30–60 °C) as the extraction solvent for 10 h and is expressed on a dry-matter basis. Crude protein content was determined using the Kjeldahl method and calculated from total nitrogen content; values are also expressed on a dry-matter basis.

### 2.9. Heavy Metal Analysis

The contents of As, Cd, Hg and Pb in larvae were determined using inductively coupled plasma mass spectrometry (ICP-MS). Samples were digested, brought to a fixed volume and analyzed for target element concentrations in the digest. Element contents in larvae were calculated from sample mass, final volume and dilution factor and are expressed as mg/kg. Technical replicate measurements were performed for each sample to provide a preliminary description of heavy metal accumulation under different kitchen waste proportions.

### 2.10. Data Processing and Statistical Analysis

Data were organized, calculated and plotted using Microsoft Excel (Microsoft Corporation, Redmond, WA, USA) and Python 3.x (Python Software Foundation, Wilmington, DE, USA). Data from treatments with three biological replicates are presented as mean ± standard error (SE). Differences among treatments were analyzed by one-way analysis of variance (ANOVA) followed by Tukey’s honestly significant difference (HSD) test, with significance set at *p* < 0.05. Principal component analysis (PCA) was performed using standardized substrate composition variables and larval response variables. Heavy metal data were based on technical replicate measurements and were therefore summarized descriptively without inferential comparison based on biological replication.

## 3. Results

### 3.1. Dynamic Changes in Individual Larval Fresh Weight Under Different Feeding Substrates

**Figure 1:**
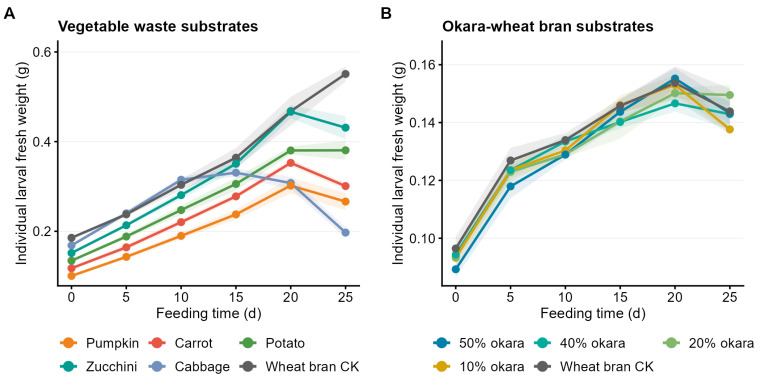
Dynamic changes in individual larval fresh weight of *Tenebrio molitor* (*T. molitor*) under different feeding substrates. (**A**) Changes in individual larval fresh weight under different vegetable waste substrates. (**B**) Changes in individual larval fresh weight under different okara substitution levels. Data are presented as mean ± standard error (SE) (*n* = 3). Individual larval fresh weight in the vegetable trial was estimated from total larval biomass, initial individual larval weight and cumulative pupal number, whereas values in the okara trial were calculated from recorded individual larval weight.

Individual larval fresh weight was used to compare larval growth under vegetable waste and okara substitution treatments. In the vegetable trial, growth patterns varied among substrates (Figure 1A). The wheat bran control increased steadily and reached the highest final individual weight (0.551 ± 0.029 g). Among vegetable substrates, zucchini resulted in higher larval weight gain than most vegetable substrates, reaching 0.467 ± 0.025 g on day 20 before declining slightly. Potato maintained a relatively high final value (0.381 ± 0.036 g). Carrot and pumpkin also reached their maxima on day 20, followed by a decline. Cabbage increased during the early stage and reached 0.331 ± 0.016 g on day 15, but then decreased sharply to 0.197 ± 0.021 g at the end of the trial, suggesting that cabbage alone may provide insufficient nutritional support for maintaining high larval biomass during later developmental stages.

In the okara substitution trial, individual larval fresh weight generally increased during the first 20 days and peaked around day 20 (Figure 1B). The highest numerical value on day 20 was observed in the 50% okara treatment (0.155 ± 0.006 g), followed closely by 10% okara and the wheat bran control (0.153 ± 0.006 g and 0.154 ± 0.010 g, respectively). The 20% and 40% okara treatments reached 0.150 ± 0.003 g and 0.147 ± 0.005 g, respectively. By the final sampling point, individual weight had declined slightly in all okara treatments. Because individual developmental stage was not directly recorded at each sampling point, this late decline should be interpreted cautiously and may reflect a combination of developmental transition, feed utilization and body water change. Overall, okara substitution did not strongly suppress individual larval growth, and 20–50% okara maintained relatively stable weights during the middle and later stages. This suggests that a substantial proportion of wheat bran could be replaced by okara without a large loss of larval fresh-weight performance under the conditions tested.

### 3.2. Effects of Vegetable Waste on Fresh-Weight-Based Conversion Efficiency and Pupal Output

**Figure 2:**
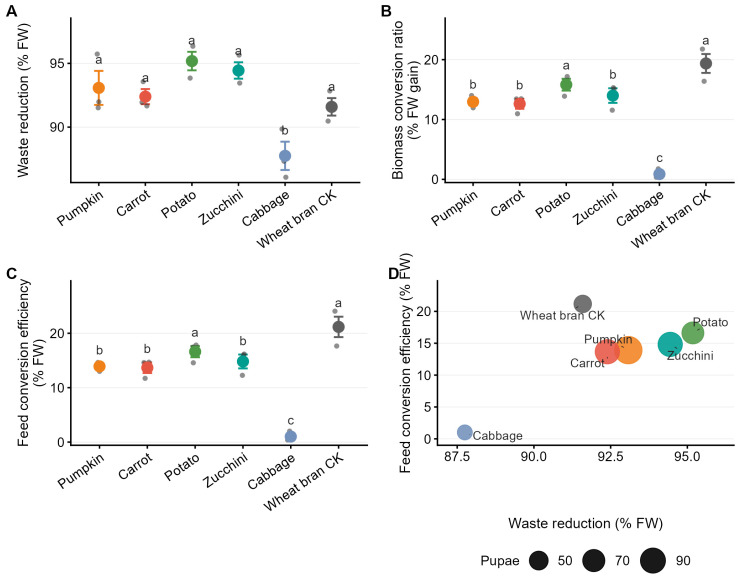
Fresh-weight-based conversion efficiency and pupal output of *Tenebrio molitor* (*T. molitor*) larvae fed different vegetable waste substrates. CK denotes the wheat bran control. (**A**) Waste reduction (WR). (**B**) Bioconversion rate (BCR). (**C**) Feed conversion efficiency (FCE). (**D**) Integrated bubble plot showing WR, FCE and pupal output; bubble size represents the mean number of pupae per replicate. Data are presented as mean ± standard error (SE) (*n* = 3). Different lowercase letters indicate significant differences among treatments based on one-way ANOVA followed by Tukey’s HSD test (*p* < 0.05). WR, BCR and FCE were calculated as fresh-weight-based operational conversion indices. The WR axes in panels (**A**,**D**) are truncated to improve visualization of treatment differences.

Vegetable substrate type affected fresh-weight-based conversion efficiency and pupal output (Figure 2). All vegetable treatments showed substantial substrate reduction. Potato showed the highest numerical WR (95.18 ± 0.73%), followed by zucchini (94.44 ± 0.64%), pumpkin (93.08 ± 1.33%) and carrot (92.39 ± 0.59%). These treatments did not differ significantly from the wheat bran control (*p* > 0.05). Cabbage had the lowest WR (87.75 ± 1.11%) and was significantly lower than the other treatments (*p* < 0.05). Thus, most vegetable substrates were effectively consumed under dynamic feeding, whereas cabbage showed weaker fresh-weight-based reduction as a sole substrate.

BCR and FCE were more sensitive to substrate type than WR. The wheat bran control had the highest BCR and FCE (19.36 ± 1.58% and 21.17 ± 1.88%, respectively). Potato also remained high, with BCR and FCE of 15.82 ± 0.99% and 16.62 ± 1.03%, respectively. Pumpkin, carrot and zucchini showed intermediate BCR and FCE values of 12.62–14.00% and 13.67–14.82%, respectively. In contrast, cabbage had significantly lower BCR and FCE (0.88 ± 0.44% and 1.01 ± 0.50%) than those of the other treatments (*p* < 0.05). This indicates that substrate disappearance in the cabbage treatment was poorly converted into larval biomass.

Pupal output further reflected the effects of vegetable substrates on developmental stability. Pumpkin produced the highest mean number of pupae (109.00 ± 8.62 pupae per replicate). Carrot and zucchini produced approximately 85 pupae per replicate and were significantly higher than potato, wheat bran and cabbage (*p* < 0.05). Although potato had high BCR and FCE, its mean pupal number was 69.00 ± 7.94 pupae per replicate. Cabbage produced the lowest pupal output (31.33 ± 3.71 pupae per replicate), indicating reduced conversion efficiency and lower pupal output rather than simply lower substrate disappearance.

The integrated bubble plot showed that vegetable substrates differed in waste reduction, conversion efficiency and developmental output (Figure 2D). Potato occupied the high-WR and high-FCE region, suggesting efficient conversion of consumed substrate into biomass. Pumpkin had slightly lower FCE than potato and wheat bran but a larger bubble size, indicating stronger pupal output. Thus, potato and pumpkin represent two different favourable substrate profiles: potato was more closely related to efficient biomass conversion, whereas pumpkin was more closely related to pupal production. Zucchini showed high WR and moderately high FCE, suggesting good overall potential. Cabbage was positioned in the low-FCE region with a small bubble size, indicating that despite some substrate reduction, it did not efficiently support biomass gain or pupation.

### 3.3. Effects of Okara Substitution Level on Fresh-Weight-Based Conversion Efficiency and Pupal Output

**Figure 3:**
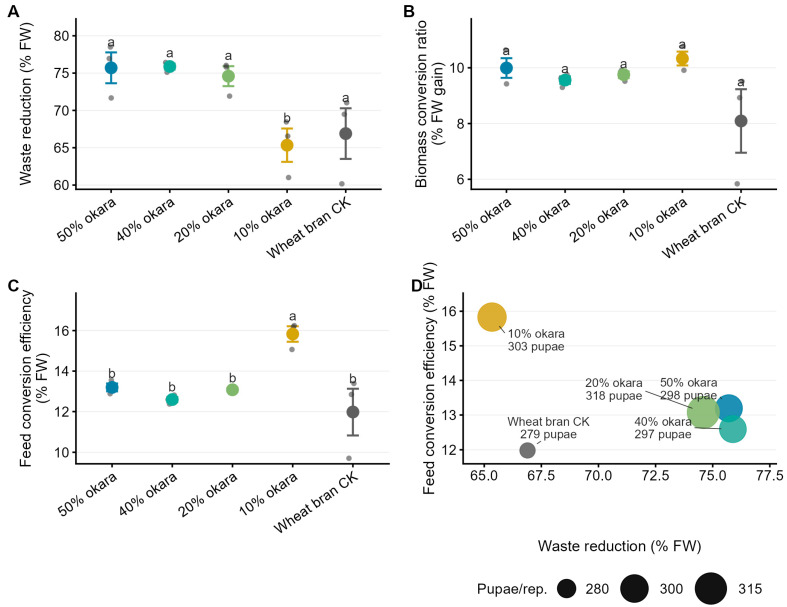
Fresh-weight-based conversion efficiency and pupal output of *Tenebrio molitor* (*T. molitor*) larvae under different okara substitution levels. CK denotes the wheat bran control. (**A**) Waste reduction (WR). (**B**) Bioconversion rate (BCR). (**C**) Feed conversion efficiency (FCE). (**D**) Integrated bubble plot showing WR, FCE and pupal output; bubble size represents the mean number of pupae per replicate. Data are presented as mean ± standard error (SE) (*n* = 3). Different lowercase letters indicate significant differences among treatments based on one-way ANOVA followed by Tukey’s HSD test (*p* < 0.05). WR, BCR and FCE were calculated as fresh-weight-based operational conversion indices. The WR axes in panels (**A**,**D**) are truncated to improve visualization of treatment differences.

Okara substitution changed substrate reduction, conversion efficiency and pupal output (Figure 3). The 40% okara treatment showed the highest numerical WR (75.88 ± 0.39%), although it was not significantly different from the 20% and 50% okara treatments (*p* > 0.05). The 50% and 20% okara treatments had WR values of 75.71 ± 2.07% and 74.59 ± 1.34%, respectively. The 10% okara treatment had lower WR (65.34 ± 2.24%) than the 20–50% okara treatments (*p* < 0.05). The wheat bran control had a WR of 66.89 ± 3.39% and did not differ from several okara treatments. These results indicate that medium to high okara inclusion is associated with greater fresh-weight-based substrate reduction.

BCR did not differ significantly among okara treatments (*p* > 0.05). The 10% okara treatment had the highest numerical BCR (10.33 ± 0.25%), followed by 50%, 20% and 40% okara (9.99 ± 0.35%, 9.75 ± 0.12% and 9.56 ± 0.14%, respectively); the wheat bran control had a BCR of 8.09 ± 1.14%. FCE responded more clearly to okara level. The 10% okara treatment had the highest FCE (15.83 ± 0.38%) and was significantly higher than the other okara treatments and the wheat bran control (*p* < 0.05). FCE values in the 20%, 50% and 40% okara treatments were 13.08 ± 0.09%, 13.19 ± 0.20% and 12.59 ± 0.13%, respectively, whereas the wheat bran control reached 11.98 ± 1.15%. Thus, lower okara inclusion favoured conversion of consumed substrate into biomass, whereas higher inclusion was mainly associated with greater substrate reduction.

Okara substitution also affected pupation. The 20% okara treatment produced the highest numerical mean number of pupae (318.33 ± 6.17 pupae per replicate). The 10%, 50% and 40% okara treatments produced 303.00 ± 6.43, 298.33 ± 4.37 and 297.33 ± 4.48 pupae per replicate, respectively. These okara treatments did not differ significantly from each other (*p* > 0.05), but were all higher than the wheat bran control (278.67 ± 0.88 pupae per replicate; *p* < 0.05). Similarly, total pupal weight was numerically highest in the 20% okara treatment (39.55 ± 0.30 g per replicate), followed by 10%, 40% and 50% okara (38.79 ± 0.70, 37.95 ± 0.50 and 37.92 ± 0.67 g per replicate, respectively), all of which were higher than the wheat bran control (35.14 ± 0.38 g per replicate; *p* < 0.05).

The bubble plot shows that different okara levels correspond to different resource-use goals (Figure 3D). The optimal okara inclusion level therefore depends on the intended production objective. Low inclusion (10%) maximized conversion efficiency, moderate inclusion (20%) improved pupal production and higher inclusion (40–50%) enhanced substrate reduction. Overall, okara substitution did not produce a single optimal level for all traits, but instead separated waste reduction, conversion efficiency and pupal output into different target responses.

### 3.4. Exploratory PCA Based on Substrate Composition and Larval Response Variables

**Figure 4:**
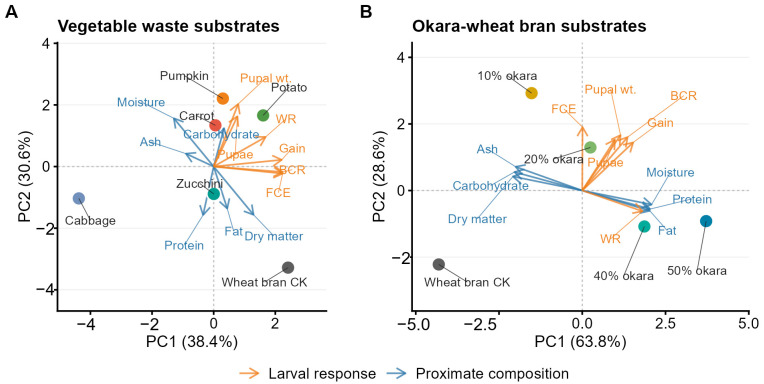
Exploratory principal component analysis (PCA) based on substrate composition and larval response variables. PC1, principal component 1; PC2, principal component 2; CK, wheat bran control. (**A**) Vegetable feeding trial. (**B**) Okara substitution trial. All variables were standardized before PCA. Composition variables included moisture, dry matter, crude protein, crude fat, ash and carbohydrates. Larval response variables included waste reduction (WR), bioconversion rate (BCR), feed conversion efficiency (FCE), biomass gain, pupal number and total pupal weight. Dashed arrows indicate proximate composition variables, and solid arrows indicate larval response variables.

PCA was performed using standardized substrate composition variables and larval response variables to visualize overall separation among treatments (Figure 4). In the vegetable feeding trial, principal component 1 (PC1) and principal component 2 (PC2) explained 38.4% and 30.6% of the total variation, respectively. Cabbage was clearly separated from the other treatments and was positioned away from the main conversion and developmental response vectors, consistent with its reduced BCR, FCE and pupal output. Potato was closer to WR and conversion-related vectors, reflecting its high waste reduction and relatively high conversion efficiency. Pumpkin was more closely associated with pupal-output variables, consistent with its higher pupal output. Zucchini was positioned nearer to fat- and moisture-related directions, whereas carrot occupied an intermediate region without a single dominant association. These results indicate that differences in vegetable substrate composition are associated with distinct larval performance patterns.

In the okara substitution trial, PC1 and PC2 explained 63.8% and 28.6% of the total variation, respectively. The 40% and 50% okara treatments were positioned closer to moisture, crude protein, crude fat and WR-related directions, indicating that higher okara inclusion was associated with greater substrate reduction. The 10% okara treatment was closer to FCE, suggesting more efficient conversion of consumed substrate into biomass. The 20% okara treatment was located near pupal-output variables, supporting its stronger developmental output. Overall, PCA supported the single-variable results and connected substrate composition with larval response variables, but it was used as an exploratory association analysis rather than as evidence of causation.

### 3.5. Effects of Kitchen Waste Proportions on Larval Nutritional Composition

**Figure 5:**
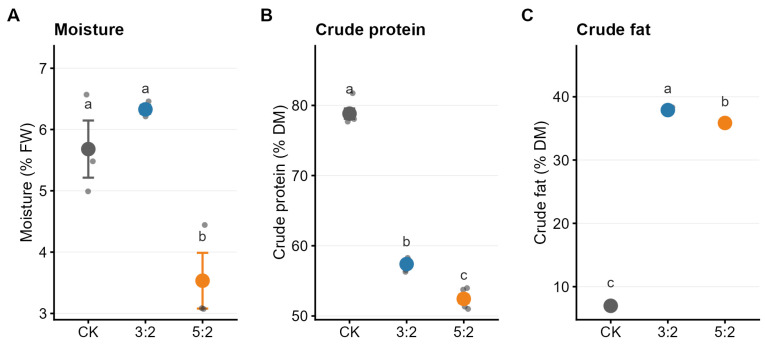
Effects of different kitchen waste proportions on the nutritional composition of *Tenebrio molitor* (*T. molitor*) larvae. CK denotes the wheat bran control. (**A**) Moisture content. (**B**) Crude protein content on a dry-matter basis. (**C**) Crude fat content on a dry-matter basis. Bars represent means, error bars indicate standard error (SE) and points represent individual replicate values. Different lowercase letters indicate significant differences among treatments based on one-way ANOVA followed by Tukey’s HSD test (*p* < 0.05). Moisture content is expressed on a fresh-weight (FW) basis, whereas crude protein and crude fat are expressed on a dry-matter (DM) basis.

Kitchen waste proportion significantly affected larval moisture, crude protein and crude fat contents (Figure 5). Moisture contents in the wheat bran control (CK) and 3:2 treatments were 5.68 ± 0.47% and 6.33 ± 0.07%, respectively, and did not differ significantly (*p* > 0.05). The 5:2 treatment decreased moisture content to 3.53 ± 0.45%, which was significantly lower than both CK and 3:2 (*p* < 0.05). This suggests that a higher kitchen waste proportion is associated with lower larval moisture content.

Kitchen waste feeding reduced the relative proportion of crude protein in larval dry matter. The CK group had the highest crude protein content (78.82 ± 0.74%). The 3:2 treatment decreased crude protein to 57.39 ± 0.40%, and the 5:2 treatment further reduced it to 52.44 ± 0.61%. All three treatments differed significantly (*p* < 0.05), indicating a continued decrease in the relative larval protein proportion as the kitchen waste level increased.

In contrast, kitchen waste markedly increased the relative crude fat proportion on a dry-matter basis. The CK group contained only 6.98 ± 0.49% crude fat, whereas the 3:2 and 5:2 treatments reached 37.90 ± 0.22% and 35.86 ± 0.16%, respectively. Both kitchen waste treatments were significantly higher than CK (*p* < 0.05), but crude fat in the 5:2 treatment was lower than that in the 3:2 treatment (*p* < 0.05), indicating that further increasing kitchen waste did not promote additional relative fat accumulation. Overall, kitchen waste inclusion altered larval nutritional composition by decreasing the relative crude protein proportion and increasing the relative crude fat proportion. The 3:2 treatment showed the most balanced composition, with high fat accumulation while maintaining higher protein content than the 5:2 treatment. However, these changes represent shifts in nutrient proportions rather than direct evidence of increased absolute nutrient yields.

### 3.6. Heavy Metal Accumulation Under Kitchen Waste Loading

**Figure 6:**
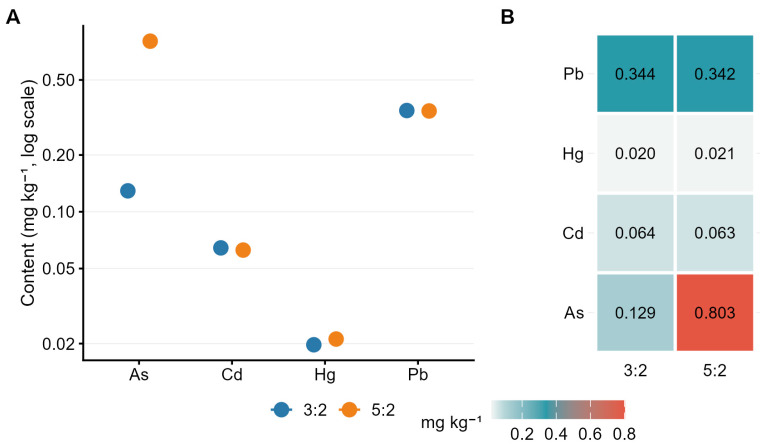
Heavy metal contents in *Tenebrio molitor* (*T. molitor*) larvae fed kitchen waste–wheat–bran mixtures. (**A**) Scatter plot of arsenic (As), cadmium (Cd), mercury (Hg) and lead (Pb) contents. (**B**) Heatmap of As, Cd, Hg and Pb contents. Blue and orange points represent the 3:2 and 5:2 treatments, respectively; heatmap cell colors show element contents. Heavy metal contents are expressed as mg/kg. Because these data were derived from technical repeated measurements, significance comparisons based on biological replicates were not performed.

To provide an initial safety screen, As, Cd, Hg and Pb contents were measured in larvae from the 3:2 and 5:2 kitchen waste treatments (Figure 6). Responses differed among elements. As was the most sensitive to increased kitchen waste loading: its content was 0.129 ± 0.001 mg/kg in the 3:2 treatment and increased to 0.803 ± 0.001 mg/kg in the 5:2 treatment, approximately 6.2 times higher than in 3:2. This indicates that higher kitchen waste inclusion may enhance As transfer from the substrate to larvae. Because these measurements were based on technical replicates and were not compared with source-specific legal thresholds or substrate concentrations, they should be interpreted as a preliminary accumulation screen rather than as a complete risk assessment.

Cd, Hg and Pb showed much smaller differences between the two kitchen waste treatments. Cd contents were 0.064 ± 0.001 and 0.063 ± 0.000 mg/kg in the 3:2 and 5:2 treatments, respectively. Hg contents were 0.020 ± 0.000 and 0.021 ± 0.000 mg/kg, and Pb contents were 0.344 ± 0.000 and 0.342 ± 0.001 mg/kg. The heatmap further shows that As is the main element separating the 3:2 and 5:2 treatments, whereas Cd, Hg and Pb are relatively similar between treatments.

## 4. Discussion

### 4.1. Interpretation Boundaries of Fresh-Weight-Based Conversion Indices Under Dynamic Feeding

This study used a dynamic feeding strategy to evaluate the ability of *T. molitor* to use multisource agri-food wastes. Compared with conventional one-time fixed feeding approaches, dynamic feeding recalculates feed input according to larval biomass at the start of each cycle. The feeding substrates and mixed diets differed markedly in moisture, dry matter and nutrient profile, which is essential for interpreting fresh-weight-based operational indices. The WR, BCR and FCE values in this study should therefore be considered together with substrate moisture and dry matter composition. This point is important because substrates with high moisture and low dry matter may show high apparent fresh-weight reduction even when conversion of dry nutrients into larval biomass is limited. Previous *T. molitor* valorization studies usually combine weight gain, feed conversion, efficiency of conversion, developmental time and survival to evaluate organic by-product use, rather than relying on a single conversion parameter [23,24]. Accordingly, the WR, BCR and FCE values reported here are most useful for comparing treatments within this experiment and should not be directly compared with dry-matter-based mass balances from other studies.

*T. molitor* is valuable as a resource insect because it can reduce low-value organic by-products and convert part of their nutrients into insect biomass for potential feed or food use. By-product diets strongly affect *T. molitor* growth, feed utilization and body composition, indicating that substrate type and composition are key factors in *T. molitor*-based valorization [23,24]. The present study therefore combined substrate proximate composition, individual fresh weight, WR, BCR, FCE, pupal number, pupal weight, larval nutritional composition and heavy metal screening to provide a more comprehensive evaluation of multisource agri-food waste suitability for *T. molitor* rearing.

### 4.2. Effects of Single Vegetable Substrates on Growth, Conversion, and Developmental Output

Single vegetable substrates differed markedly in their effects on individual larval fresh weight, fresh-weight-based conversion efficiency and pupal output. Potato showed the highest numerical WR and maintained relatively high BCR and FCE, suggesting that it was both readily consumed and efficiently converted into larval biomass. Zucchini also showed high WR and moderately high FCE. Pumpkin did not have the highest FCE but produced the greatest pupal output, suggesting that it supported transition to the pupal stage. Fresh plant materials and vegetable waste supplements have been reported to affect *T. molitor* growth and nutrient composition, and may function as moisture and nutrient supplements in rearing systems [14,25]. This agrees with the ability of several vegetable substrates in the present study to support larval growth and developmental output.

Cabbage, however, showed a clear mismatch among waste reduction, conversion and development. Although cabbage still showed some WR, its BCR, FCE and pupal output were very low. Thus, apparent substrate disappearance did not translate into effective biomass formation. This may be related to the high moisture, low dry matter and low usable nutrient density of leafy vegetable substrates. Cabbage also has a watery tissue structure and relatively low energy density, which may limit sustained intake of dry nutrients even when fresh material disappears from the feeding tray. Such substrates can be ingested and may show apparent reduction in a short-term trial, but if dry matter, protein or available energy is insufficient, the ingested material may not support sustained growth or metamorphosis. Studies on agro-industrial diets also show that by-products differ strongly in their effects on *T. molitor* production, digestibility and body composition; ingestion alone therefore does not guarantee efficient conversion [8,9].

These findings indicate that vegetable waste suitability should not be judged by WR alone. For high-moisture or low-dry-matter substrates, residue disappearance may overestimate resource value. Evaluation of single vegetable substrates should include individual larval weight, BCR, FCE, pupal number and pupal weight. Based on the current data, potato, zucchini and pumpkin showed greater potential for vegetable waste valorization, whereas cabbage alone may provide insufficient nutritional support for maintaining high larval biomass and pupal output during later developmental stages.

### 4.3. Target Differentiation Among Waste Reduction, Conversion Efficiency and Pupal Output Under Okara Substitution

The okara substitution trial further showed that compound substrate optimization does not identify a single best proportion across all indicators. Instead, different okara levels favoured different objectives. Treatments containing 20–50% okara generally had high WR, with 40% okara showing the highest numerical reduction. The 10% okara treatment had the highest FCE, indicating more efficient conversion of consumed substrate into biomass. The 20% okara treatment produced the greatest number and mass of pupae, indicating stronger developmental output. These differences show that okara inclusion should be interpreted according to the intended production target rather than as a single linear dose response.

Okara contains protein, lipids and fibre, and its inclusion may improve the nutrient profile of wheat bran-based diets. At moderate levels, okara may supplement moisture and nutrients while maintaining the physical structure provided by wheat bran. At high levels, however, okara may also change moisture, aeration, looseness or fibre load, thereby affecting feeding, digestion and development. This may explain why higher inclusion was associated with stronger fresh-weight substrate reduction, whereas lower or moderate inclusion was more favourable for FCE or pupal output. In black soldier fly systems, mixed substrates such as okara with kitchen waste or dairy manure can alleviate the nutritional or physical limitations of single waste streams [1,26]. Although black soldier fly and *T. molitor* differ in feeding behaviour, developmental timing and substrate adaptation, the broader principle that substrate blending can reduce single-substrate limitations is relevant here.

In *T. molitor* rearing, food by-products and agro-industrial residues can also influence growth, body composition, microbial load and antioxidant status [12,27]. The choice of okara inclusion level should therefore depend on the intended production target. If the goal is waste reduction, approximately 40–50% okara deserves attention. If the goal is conversion efficiency, a lower inclusion level may be more suitable. If the goal is stable pupal output, the 20% okara treatment appears worthy of further testing. The relatively stable larval fresh weight under 20–50% okara also suggests that a substantial proportion of wheat bran could be replaced by okara without a large loss of larval fresh-weight performance. In practical terms, the best compound diet should be selected by multi-indicator evaluation rather than by WR or FCE alone.

### 4.4. Nutritional Remodelling of Larvae by Kitchen Waste Mixture Substrates

Kitchen waste mixtures substantially altered larval nutritional composition. Compared with the wheat bran control, both the 3:2 and 5:2 kitchen waste treatments increased the relative crude fat proportion on a dry-matter basis while lowering the relative crude protein proportion. This suggests a shift from protein-rich, low-fat biomass towards a higher-fat larval product, but it does not by itself demonstrate increased absolute fat or protein yield. *T. molitor* body composition is known to respond strongly to diet; agricultural sidestreams, by-products and moist supplements can alter protein, lipid, fatty acid, mineral and heavy metal profiles [18,22]. The fat increase and protein decrease observed in the present kitchen waste treatments are consistent with this substrate-driven plasticity.

The 3:2 treatment produced high relative crude fat while retaining a relatively higher crude protein level, whereas increasing kitchen waste to 5:2 further decreased crude protein without increasing crude fat. Thus, the relationship between kitchen waste proportion and larval fat deposition is not simply linear. Excessive kitchen waste may unbalance substrate nutrients or modify palatability, aeration and the microenvironment, reducing protein deposition efficiency. *T. molitor* is sensitive to protein, carbohydrate and lipid balance, and dietary macronutrient ratios affect growth, protein content and fat accumulation [11,15].

From an application perspective, kitchen waste feeding can change the target use of *T. molitor* biomass. If a high-protein insect meal is desired, the kitchen waste proportion should be controlled to avoid excessive protein dilution. If a higher-fat biomass or insect lipid fraction is the target, moderate kitchen waste inclusion may be useful. In the present study, the 3:2 kitchen waste:wheat bran ratio appeared to provide a better balance between increased relative fat content and protein retention, whereas the 5:2 ratio may carry greater nutritional imbalance and a stronger need for safety verification.

### 4.5. Heavy Metal Accumulation Risk and Safety Boundaries of Kitchen Waste Feeding

Safety is a key constraint when insects reared on waste streams are intended for feed or food use. In the present heavy metal screen, As responded most strongly to increased kitchen waste proportion, whereas Cd, Hg and Pb changed only slightly between the 3:2 and 5:2 treatments. The As content in the 5:2 treatment was approximately 6.2 times that in the 3:2 treatment, indicating that As may be a more sensitive element for substrate-to-larva transfer under the kitchen waste conditions tested here. Previous studies have shown that *T. molitor* and black soldier fly can take up Cd, Pb and As from contaminated substrates, and that element transfer differs among metals [28]. Feeding substrate also influences the presence of Cd, Pb, Ni, As and Hg in *T. molitor* larvae and the associated consumption risk [29].

As with nutrient composition, contaminant risk is substrate-source dependent. Kitchen waste is a complex stream, and heavy metal input can vary with raw materials, processing, tableware contact, seasonings, cooking residues and collection batch. Beyond heavy metals, insects reared on waste substrates may face risks related to pesticide residues, mycotoxins and other chemical contaminants [30,31]. Feedstock composition can also affect larval nutritional profiles and process performance [32,33]. *T. molitor* valorization studies should therefore treat product safety as a core endpoint rather than as an ancillary observation.

The heavy metal data in this study were based only on technical replicate measurements and did not include independent biological replicates or multiple kitchen waste batches. They should therefore be interpreted as preliminary screening rather than as evidence for a dose–response pattern, legal compliance or industrial safety threshold. Future work should measure heavy metals in the substrate, larvae and frass, and calculate enrichment or transfer factors to clarify the uptake, accumulation and excretion of As, Cd, Hg and Pb. Microbial safety, pesticide residues, mycotoxins and other contaminants should also be incorporated into a more complete safety framework for kitchen waste-based *T. molitor* rearing.

### 4.6. Integration of Substrate Composition and Larval Response Variables by PCA

The revised PCA provided a compact visualization of both substrate composition variables and larval response variables. In the vegetable trial, the separation of cabbage from the other treatments was associated with weak conversion and developmental performance, whereas potato and pumpkin were more closely related to conversion- and pupal-output vectors, respectively. Zucchini was closer to fat- and moisture-related directions, and carrot occupied an intermediate position without a clear dominant relationship. In the okara trial, higher okara inclusion levels were associated with moisture, crude protein, crude fat and WR-related directions, whereas the 10% okara treatment was closer to FCE and the 20% okara treatment was closer to pupal-output variables. These patterns support the single-variable analyses and indicate that differences in substrate composition and inclusion level are associated with distinct larval performance patterns.

Although inclusion of substrate composition variables improves the interpretation of treatment separation, PCA should still be regarded as an exploratory visualization rather than a causal model. The number of treatments was limited, and the analysis was intended to summarize multivariate patterns rather than identify definitive nutritional drivers. Therefore, the PCA helps connect substrate composition with larval response variables, but it does not prove that a single nutritional component caused a specific performance outcome. Future studies with more substrate batches, direct dry-matter mass balance and larger treatment numbers could further use PCA, partial least squares regression, redundancy analysis or correlation networks to link substrate composition with conversion efficiency, product quality and safety screening.

### 4.7. Limitations and Future Directions

This study has several limitations. First, WR, BCR and FCE were calculated on a fresh-weight basis. These indices describe operational conversion under the dynamic feeding regime but cannot replace a complete dry-matter mass balance. Although substrate proximate composition was determined, dry matter flow among feed input, residues, frass and larval biomass was not fully established. Therefore, the conversion indices should be interpreted together with substrate moisture and dry matter composition. Second, the kitchen waste nutritional and heavy metal analyses were preliminary, especially because heavy metal screening lacked independent biological replication, multiple kitchen waste batches, substrate metal concentrations and comparison with relevant safety thresholds. Third, the present study did not include amino acid profiles, fatty acid profiles, microbial safety or contaminant-transfer coefficients, which would be needed for a more complete evaluation of the feed and safety value of *T. molitor* biomass.

Further work should combine dry-matter-based mass balance with substrate composition, frass nutrients, amino acid profiles, fatty acid profiles, mineral elements, microbial safety and contaminant transfer. Such data would provide a more complete evaluation of *T. molitor*-based conversion of multisource agri-food wastes. The 20–40% okara substitution range and the 3:2 kitchen waste–wheat–bran mixture should be tested further with more substrate batches, biological replicates and longer rearing periods to evaluate effects on growth, pupation, emergence, reproduction, absolute nutrient yields and product quality.

## 5. Conclusions

*T. molitor* responses to multisource agri-food wastes were strongly substrate-dependent. Substrate composition, especially moisture, dry matter and nutrient profile, helped explain differences in fresh-weight-based conversion and developmental output. Among single vegetable substrates, potato, zucchini and pumpkin showed greater reduction and conversion potential, whereas cabbage showed a clear mismatch between substrate disappearance, biomass formation and pupal output. Okara substitution improved the use of compound substrates, but different inclusion levels favoured different goals. Kitchen waste mixtures increased the relative larval fat proportion, but high kitchen waste loading was associated with lower relative protein content and greater larval arsenic (As) content in the preliminary technical-replicate screen. *T. molitor*-based agri-food waste valorization should therefore consider substrate composition, reduction efficiency, biomass conversion, developmental stability, nutritional composition and safety screening together, rather than focusing only on waste reduction.

## Data Availability

The data supporting the findings of this study are available from the corresponding author upon reasonable request.
